# Mouse polyQ database: a new online resource for research using mouse models of neurodegenerative diseases

**DOI:** 10.1186/s13041-015-0160-8

**Published:** 2015-10-29

**Authors:** Wojciech J. Szlachcic, Pawel M. Switonski, Małgorzata Kurkowiak, Kalina Wiatr, Maciej Figiel

**Affiliations:** Institute of Bioorganic Chemistry, Polish Academy of Sciences, Noskowskiego 12/14, 61-704 Poznań, Poland

**Keywords:** Polyglutamine diseases, Mouse models, Database, Neurodegenerative disease, Therapy, Huntington disease, Spinocerebellar ataxia, DRPLA, SBMA

## Abstract

**Background:**

The polyglutamine (polyQ) family of disorders comprises 9 genetic diseases, including several types of ataxia and Huntington disease. Approximately two decades of investigation and the creation of more than 130 mouse models of polyQ disorders have revealed many similarities between these diseases. The disorders share common mutation types, neurological characteristics and certain aspects of pathogenesis, including morphological and physiological neuronal alterations. All of the diseases still remain incurable.

**Description:**

The large volume of information collected as a result of the investigation of polyQ models currently represents a great potential for searching, comparing and translating pathogenesis and therapeutic information between diseases. Therefore, we generated a public database comprising the polyQ mouse models, phenotypes and therapeutic interventions tested in vivo. The database is available at http://conyza.man.poznan.pl/.

**Conclusion:**

The use of the database in the field of polyQ diseases may accelerate research on these and other neurodegenerative diseases and provide new perspectives for future investigation.

## Background

Polyglutamine (polyQ) diseases are evoked by a special type of mutation whereby the expansion of CAG trinucleotide repeats in coding exons of causative genes occurs. Nine diseases with this type of mutation have been identified, including Huntington disease (HD) [1]; spinocerebellar ataxia (SCA) type 1 [2], type 2 [3], Machado-Joseph disease (MJD/SCA3) [4], type 6 [5], type 7 [6], and type 17 [7, 8]; dentatorubral pallidoluysian atrophy (DRPLA) [9, 10]; and spinal and bulbar muscular atrophy (SBMA) [11, 12]. All of these conditions are neurological disorders that share several commonalities, including similar mutation genetics and numerous aspects of pathogenesis. All of these disorders are rare. However, these conditions are highly significant for understanding pathogenic mechanisms in neurodegeneration because the defined etiology of polyQ diseases induces a spectrum of neurological symptoms in patients. Enormous interest in polyQ diseases has led to the generation of more than 130 mouse models, and investigation of these models has helped define polyQ pathogenesis. Therefore, the research continues with new models being generated and published [13, 14].

The neurological features of polyQ disorders in patients include, motor abnormalities, such as gait ataxia, sensory defects, ocular, speech, cognitive and other symptoms. All symptoms become evident in the third or fourth decade of life and the severity is mainly dependent on length of the CAG expansion and other genetic modifiers. The mechanisms of pathogenesis in polyQ diseases have been discussed also recently [15–18]. In brief, the relevant pathogenesis is based on the toxic function of the mutant polyQ proteins. Long polyglutamine domain in causative proteins misfolds and aggregates into cytoplasmic and nuclear insoluble inclusions. Both inclusions and soluble polyglutamine domains interact with various cellular components exerting a spectrum of cellular dysfunction including transcriptional deregulation, mitochondrial dysfunction, clearance machinery impairment, increased susceptibility to excitotoxicity, inflammation and oxidative damage, and apoptosis induction [19, 20]. The elucidation of these mechanism was possible because of generation and investigation of the animal models, creating enormous data volume about pathogenesis and therapy which was the main requirement for creation of dedicated database.

Mice as model organisms are small, easy to breed and available in different strains, thus constituting a cost-effective genetic platform. Various biotechnology tools are available to modify the mouse genome, including embryonic stem cells, vectors and recent gene editing tools, such as zinc-finger nucleases (ZFNs), transcription activator-like effector nucleases (TALENs) and clustered regulatory interspaced short palindromic repeats (CRISPRs) [21, 22]. Finally, there are also various online resources providing information about the biology of the mouse, including the complete sequence of the mouse genome, gene expression atlases in various tissues, mouse anatomy atlases and databases of mouse behavior. The best known resources include the Mouse Genome Informatics database (http://www.informatics.jax.org/) [23], Allen Mouse Brain Atlas (http://www.brain-map.org) [24], EBI Gene Expression Atlas (http://www.ebi.ac.uk/gxa/home) [25], Ensembl (www.ensembl.org) [26], NCBI online services (http://www.ncbi.nlm.nih.gov) [27] and many others.

We previously generated electronic resources in the form of Excel-based databases/tables containing more than 4000 records describing the pathogenesis and therapeutic approaches in polyQ mouse models [19, 20]. We now present an online public database that contains updated polyQ mouse model data in a format that is easy to search, compare and integrate the information across polyQ models.

## Results

### Database content

**Fig. 1:**
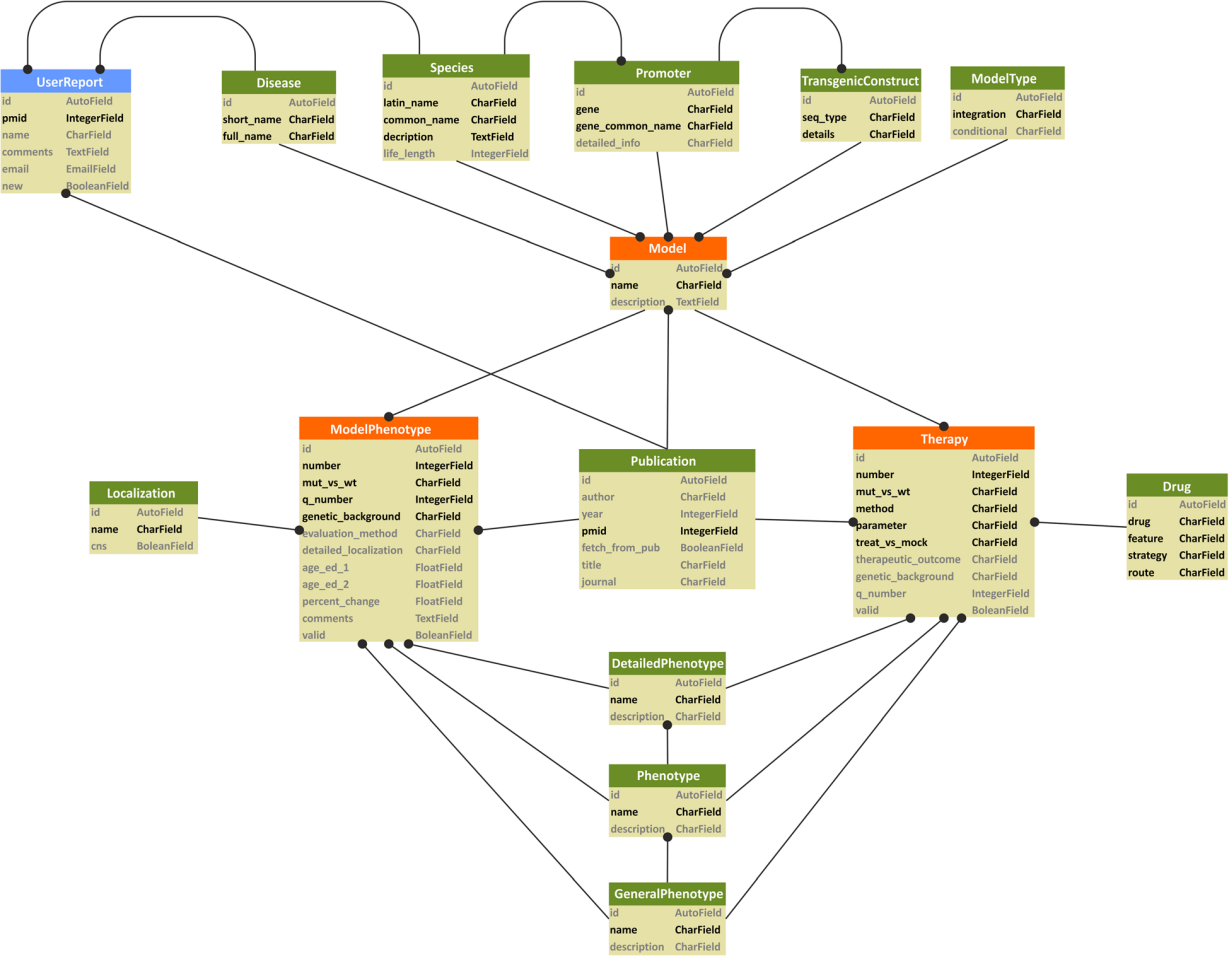
The diagram illustrates the structure of tables in the database. The main orange tables are “Model”, “ModelPhenotype” and “Therapy”. The connections with circles indicate the source of data supply. The gray and black colors in tables indicate optional and mandatory values respectively and reflect the way in which the potential records in the back end of the database are generated. Potential record cannot be generated unless all mandatory fields (black) are not empty. In turn record can be generated also without “gray” (optional) fields

The most recent version of the database comprises data on the mouse models of 9 polyQ diseases. It contains basic information about the mouse models, such as their genetic design, and detailed descriptions of the disease phenotypes. Moreover, the database contains information regarding therapeutic approaches evaluated in polyQ mice, including information about drugs and the phenotypes that were evaluated as therapeutic outcomes. Figure 1 presents the current structure of the database (see Materials and methods). Table 1 presents the overall number of records for selected tables and short description of the records.

**Table 1:** Database content

Database tables (names not visible for end user)	Number of records	Description
Models	135	Mouse models of polyQ diseases
Drugs	294	Small molecule drugs or experimental paradigms (silencing, overexpression or co-expression of molecules) assessed in preclinical therapeutic trials using polyQ models
ModelPhenotypes	2579	The number represents the individual phenotypic records of the 135 mouse models
Therapy	2380	The number represents the individual therapeutic records, i.e., phenotypes assessed therapeutically for improvement or deterioration of the response to drugs or therapeutic paradigms
Publications	669	Total number of publications analyzed to evaluate phenotypes and therapeutic approaches

### Online interface and query of the database

**Fig. 2:**
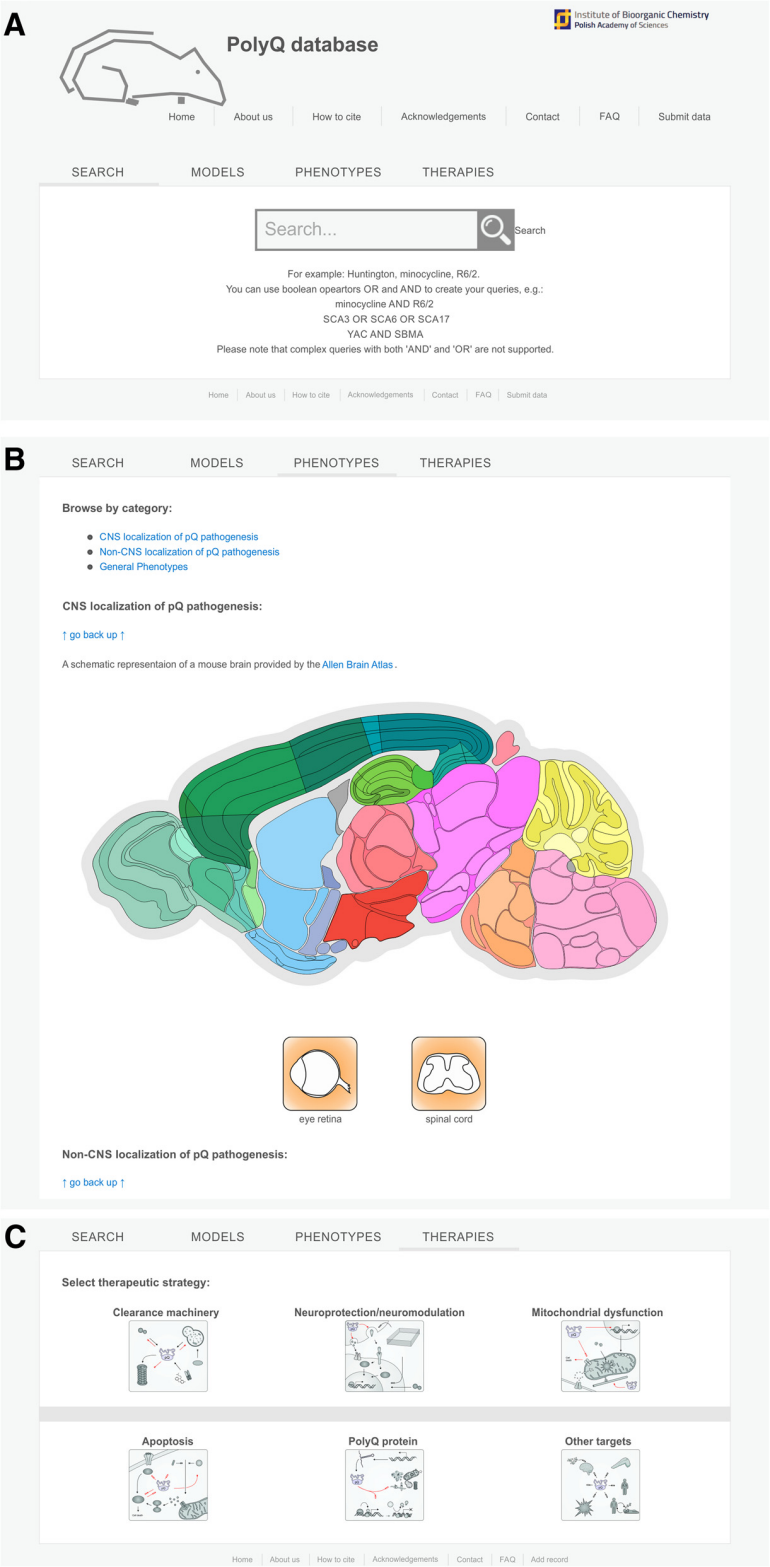
The online interface to the database and search mechanisms. **a** The interface includes a “Search” tab and three thematic tabs namely “Models”, “Phenotypes” and “Therapies” to display and group the search results with respect to models, phenotypes and therapies. The database contains an intuitive search interface to easily obtain search results. The intuitive search offers simple clicking on pictograms to find (**b**) CNS and non-CNS localization of the phenotypes and (**c**) therapeutic strategy

The online interface to the database is organized as a web page with a “Search” tab and three thematic tabs entitled “Models”, “Phenotypes” and “Therapies” (Fig. 2a). The three thematic tabs in the online interface are designed to present and group the search results with respect to models, phenotypes and therapies.

Using the “Search” tab, the user may perform a classic text search, and the results are displayed in the remaining three thematic tabs “Models”, “Phenotypes” and “Therapies”. Instead of performing the text search, the user may also opt to perform an “intuitive” search using lists and pictograms available in the “Models”, “Phenotypes” and “Therapies” tabs (Fig. 2b,c). The intuitive search is performed by selecting the disease and most common mouse models from the list in tab “Models”, selecting the phenotype location on the brain diagram (Lein et al. [24]) and pictograms of other central nervous system (CNS) and non-CNS organs and tissues in tab “Phenotypes” (Fig. 2b) and selecting from more than 20 therapeutic strategies using 6 icons, which schematically group the strategies (Fig. 2c).

### Keywords to perform a classical text search of the database

To search the database, several types of inputs can be used, including the diseases (e.g., “HD”, “SCA3”), common name of model (e.g., “R6/2”, “Ki91”), detailed phenotype (e.g., “inclusions”, “incoordination”), location (e.g., “cortex”, “cerebellum” or “liver”), detailed location (e.g., “Purkinje”), drug (e.g., “minocycline”) and therapeutic strategy (e.g., “apoptosis”, “cell therapy”). Keywords can be constructed using Boolean operands “AND”, “OR” and “NOT” which can be used several times in one query or used in complex queries containing both “AND”, “OR” and NOT. Original publications can be accessed by searching their PMID or the first author of the publication.

Search process ultimately yields the search results in thematic tabs that offer methods to refine the search results through selections from drop-down lists. Selecting a record in any of the thematic tabs results in the display of a detailed mouse model page, which contains a model name as a header and is focused on a particular model and either phenotypes or therapies. If both therapies and phenotypes are available for the given model, the detailed mouse model page allows switching between the phenotypic and therapeutic views.

### Examining the search results in the “Models”, “Phenotypes” and “Therapies” tabs

**Fig. 3:**
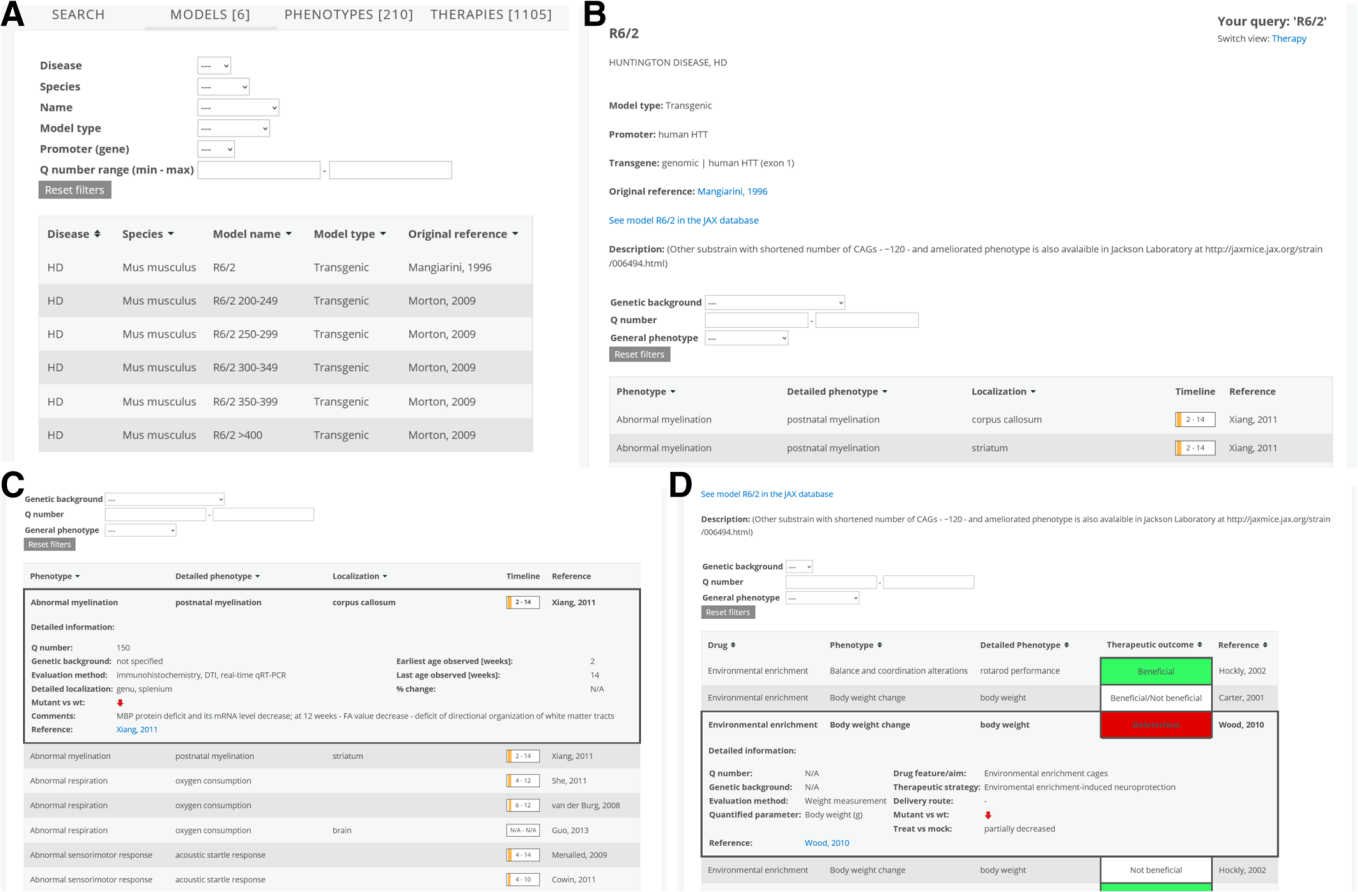
The “Models”, “Phenotypes” and “Therapies” thematic tabs. **a** Search results are available in all thematic tabs and the tab “Models” is displayed first (default setting) revealing a list of mouse models. The search can be further refined using dropdown lists. **b** Detailed mouse model page in the phenotype mode is displayed after selecting a model in the “Models” tab or a phenotype in “Phenotypes” tab. **c** The detailed mouse model page contains a list of phenotypes that can be expanded to visualize the detailed box for each phenotype. **d** Selecting a therapeutic approach in “Therapies” tab will open a detailed mouse model page in the “therapeutic mode”. Each therapeutic record from the list can be expanded to visualize the box with detailed information

The “Models” tab in the online interface mostly collects data from “Model” and “Model Phenotype” tables present in the structure of the database. After performing the text or intuitive search, the “Models” tab will appear first and present the range of mouse models (Fig. 3a). From within the “Models” tab, the user is able to select a model, which will present a detailed mouse model page (Fig. 3b) with basic information about the particular mouse model, such as mouse model name, modeled disease, model type, promoter, transgene and the reference of the original description of the model. The page with model details also contains information about genetic background, number of polyQ, an external link to the JAX Mice Database webpage and, most importantly, the phenotypes or therapeutic approaches in the model. For instance, in the R6/2 detailed mouse model page, the user can find that this is a transgenic HD model that contains the human HD promoter and expresses the N-terminal fragment of human huntingtin (HTT) protein with a 120 polyQ stretch.

The “Phenotypes” tab collects data from the “Model Phenotype” table found in the structure of the database. The records displayed in the “Phenotypes” tab provide an overview of the manifestation of the disease in a mouse model. The phenotypes in the database are subcategorized into four main types: “cognitive”, “motor”, “neuropathology” and “other”. Phenotypes in these groups are further subdivided into “phenotype” and “detailed phenotype”, which provide additional levels of phenotype complexity and enabling a methodical incorporation into the database. The above structure of the phenotype description is reflected in the “Phenotypes” tab. The records presented in the “Phenotypes” tab show phenotype manifestation in a particular model, its details, disease, model name and the reference. The search can further be refined using parameters, such as location of the phenotype in tissues selectable from dropdown lists. Selecting a model in “Phenotypes” tab opens a detailed mouse model page in the “phenotype mode” (Fig. 3b). The detailed mouse model page in this mode provides a list of phenotypes, which can be expanded or collapsed again to visualize or hide the detailed description box for each phenotype. The detailed box for each phenotype (Fig. 3c) contains a graphical representation of the age of mice when the phenotype is manifested, the information about the decreased or increased phenotype in mutant mice compared with wild type (WT) mice, number of repeats in the transgene (Q number), genetic background of mice and the Pubmed link.

The tab “Therapies” summarizes the therapeutic approaches using data from “Therapy” table found in the structure of the database. The therapeutic approaches listed in the “Therapies” tab contain the therapeutic strategy, drug that was applied to the mouse model, disease, model name and the reference. Similar to other thematic tabs, selecting a therapeutic approach will open a detailed mouse model page in the “therapeutic mode”. The detailed mouse model page in this mode provides a list of therapeutic records, each containing a drug, the respective mouse phenotype and detailed phenotype that was affected by the drug and the color-coded therapeutic outcome. Each therapeutic record from the list can be expanded or collapsed to visualize or hide the detailed box. The detailed box for each therapeutic record contains details, such as drug feature or aim, delivery route, therapeutic strategy, method of quantification of the therapeutic effect, effect on this phenotype, and description of the effect by listing whether “mutant vs. WT” and “treat vs. mock” was “decreased” or “increased” (Fig. 3d).

### Interaction with users

The database also contains a dedicated “submit data” page where research publication from Pubmed containing mouse models can be submitted to the database for further verification by database administrators. The dataset of the database is also available for research users upon request by e-mail to mfigiel@ibch.poznan.pl. In addition, the database is open for models of other neurological diseases, such as Alzheimer and Parkinson, and also for other model species.

## Discussion

Researchers in the polyQ field have generated and published 135 polyQ mouse models for 9 diseases over a period of approximately 20 years. The research over the years has been a great success and reflects the intensity of gaining a better understanding of the pathogenesis of polyQ diseases from an average of 7 models per year. As a result, numerous therapeutic strategies are now being tested for these diseases. The aforementioned volume of data available regarding the pathogenesis and preclinical therapies of polyQ diseases was the motivation behind the generation of this database. Therefore, we created one comprehensive digital repository of polyQ mouse models. This repository enables both broad and selective exploration of data on existing models, phenotypes and experimental therapeutic approaches for polyQ neurological diseases. Another motivation in generating a dedicated database was the fact that all polyQ diseases share important similarities. The most basic shared features are their common mutation type, neurological characteristics and the fact that several polyQ diseases are indistinguishable based only on the clinical features of patients and the phenotypes in animal models. Moreover, the diseases share commonalities in gene expression profiles; degeneration of common cell types in the brain, such as Purkinje cells; or even commonalities with other neurological diseases, such as accumulation of amyloid beta and tau protein [28, 29]. Therefore, the study of a polyQ disease may shed light on the pathogenic mechanism of other neurologic disorders, and this database may serve as an appropriate tool to achieve this aim.

Mouse model database has previously been represented by Mouse Genome Informatics (MGI) databases [23] and The International Mouse Phenotyping Consortium [30, 31]. In addition, a database of models available from a repository is maintained by The Jackson Laboratory (http://www.jax.org/). The MGI database is a valuable resource providing a digital repository of mouse models of all known human diseases, including the polyQ disorders. The function of the MGI database is to provide a global overview of diseases and models. Therefore, our database is a response to the need of a smaller, more detailed and focused, manually curated database. For instance, the increased “resolution” of our database is reflected in the detailed description, such as the tissue location of the phenotype, direct access to the reference, age of the animal and how the phenotype was altered compared with the WT animal. Another innovative aspect of the database that is not found elsewhere is the availability of the therapeutic approaches and strategies to combat polyQ disorders. Additionally, the therapeutic section presents a higher level of details indicating whether the therapy was beneficial, not beneficial or deleterious.

## Conclusions

Taken together, the polyQ model database provides the latest and most detailed phenotypic and therapeutic information about polyQ mice. Therefore, we anticipate that this database will help in elucidating the disease mechanisms and planning new effective treatments.

## Materials and methods

### Software tools

Previously published Excel data tables were used as the initial source of data for the database [19, 20]. The database was generated using the open source PostgreSQL 9.1.13 database engine (The PostgreSQL Global Development Group, http://www.postgresql.org). Direct access to the database is possible at the level of Apache2 server (The Apache Software Foundation, Forest Hill, MD, USA; http://www.apache.org/). The internet application was created using Python 2.7.3 language with the use of appropriate libraries (https://pypi.python.org/pypi) (The Python Software Foundation; https://www.python.org/). Searching of the database contents is provided by implementation of Elastic browser version 0.90.10 (Elasticsearch BV, Mountain View, CA, USA; https://www.elastic.co).

### Structure of the database

The structure of the database is based on 15 tables (Fig. 1). The core consists of the 3 most important tables named “Model”, “Model Phenotype” and “Therapy”. The “Model” table describes the mouse model and collects data from tables “Disease”, “Species”, “Promoter”, “Transgenic Construct”, “Model Type” and “Publication”. The “Model” table delivers the collected data to the “Model Phenotype” and “Therapy” tables. Both tables are supplied with data about the phenotypes from 3 tables named “General Phenotypes”, “Phenotypes” and “Detailed Phenotypes”.

Currently, the data in our database are limited to polyQ diseases; however, the structure of the database has been built to include other model species, such as rat, ape, sheep, and cat (“Species” table), and other diseases, such as Alzheimer or Parkinson Disease (“Disease” table), without further adjustments.

## Abbreviations

CNS: Central nervous system
HD: Huntington disease
MGI: Mouse genome informatics
polyQ: Polyglutamine
SCA: Spinocerebellar ataxia
WT: Wild type
